# Extracorporeal Lung Support as a Bridge to Diagnosis of Pulmonary Tumor Embolism

**DOI:** 10.1155/2016/3257084

**Published:** 2016-12-14

**Authors:** Vishnu Vasanthan, Kieran Halloran, Lakshmi Puttagunta, Jayan Nagendran

**Affiliations:** ^1^Department of Surgery, University of Alberta, Edmonton, AB, Canada; ^2^Mazankowski Alberta Heart Institute, Edmonton, AB, Canada; ^3^Department of Medicine, University of Alberta, Edmonton, AB, Canada; ^4^University of Alberta Hospital, Edmonton, AB, Canada

## Abstract

Bridging to diagnosis is an emerging technique used in end-stage cardiorespiratory failure that prolongs a patient's life using various modalities of extracorporeal lung support (ECLS) to achieve antemortem diagnosis. Pulmonary tumor embolism occurs when cell clusters travel from primary malignancies through venous circulation to the lungs, causing respiratory failure through inflammatory and venoocclusive pathways. Due to its nonspecific symptomatology, pulmonary tumor embolism remains an elusive diagnosis antemortem. Herein, we bridge a patient who presented in acute respiratory failure to the diagnosis of pulmonary tumor embolism from a gastric signet-ring cell carcinoma using ECLS modalities including venoarterial extracorporeal membrane oxygenation and centrally cannulated Novalung pumpless extracorporeal lung assist. We demonstrate the utility of this approach in diagnostically uncertain cases in unstable patients who are potentially acceptable ECLS and transplant candidates.

## 1. Introduction

In end-stage cardiorespiratory failure, patients can be placed on various modalities of extracorporeal lung support (ECLS) as a bridge to recovery [1], transplantation [2], or diagnosis [3]. While bridging to recovery or transplantation is employed after achieving diagnosis, bridging to a diagnosis is a strategy of prolonging a patient's life to identify the cause of cardiorespiratory failure. As a patient is bridged to diagnosis, there is opportunity to assess and evaluate suitability for potential recovery, transplantation, or appropriate withdrawal of support.

Pulmonary tumor embolism occurs when clusters of cells from a primary malignant tumor invade venous circulation and travel to the lungs. This can result in pulmonary hypertension by mechanical obstruction, production of microthrombi from coagulation cascades, and induction of concentric hypertrophy via inflammatory pathways [4–6]. Pulmonary hypertension in the context of lung carcinomatosis was first described by Bristowe in 1868 [7]. Pulmonary tumor embolism was first documented by Schmidt [8], and Kane et al. [9] used autopsy studies to report multiple tumor emboli as a cause of dyspnea. Despite previous reports, the nonspecific symptomatology makes pulmonary tumor embolism an elusive diagnosis antemortem [6].

Herein, we report the use of venoarterial extracorporeal membrane oxygenation (VA-ECMO) and Novalung pumpless extracorporeal lung assist (pECLA) as a bridge to diagnosis of pulmonary tumor embolization secondary to gastric signet-ring cell carcinoma.

## 2. Case Presentation

**Figure 1:**
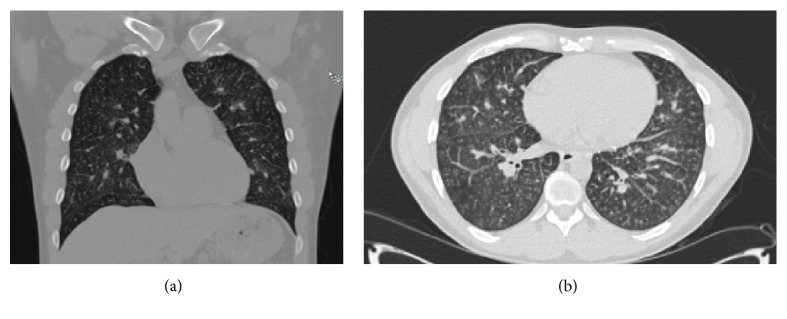
Coronal (a) and transverse (b) computed tomography views of the chest on first presentation. Images show ground-glass centrilobular micronodularities with perihilar ground-glass opacities. There is mild septal thickening and clear airways. Pulmonary artery is 3.3 cm, suggesting pulmonary hypertension.

A 38-year-old previously healthy male (Table 1) presented with 2 months of MRC grade 3 dyspnea and 9 kg weight loss with no fever or night sweats. On arrival to the emergency room, he was requiring 6 L/min oxygen via nasal prongs to maintain saturations greater than 90%. Chest X-ray and chest computed tomography (CT) (Figure 1) suggested possible pneumonia overlying interstitial lung disease and pulmonary hypertension. CT and ultrasound showed small left axillary lymph nodes unamenable to biopsy. Abdominal ultrasound was grossly normal. Thus, the patient was diagnosed with presumed pneumonia and started on antibiotics, antiviral agents, inhaled corticosteroid, and bronchodilator therapy, with enoxaparin for venous thromboembolism prophylaxis.

Over the next 5 days, the patient's oxygen saturation was kept above 90% with 8–10 L/min oxygen via nonrebreather mask. Barriers to diagnosis included inability to produce sputum despite induction, high oxygen demands contraindicating bronchoscopy, small lymph nodes unamenable to biopsy, and intolerance of supine position needed for perfusion scans. The patient was transferred to the Thoracic Surgery service for a surgical lung biopsy on day 6. However, the patient experienced right-sided chest pain, diaphoresis, and tachycardia of 160 beats per minute, followed by a convulsive episode with no postictal symptoms. After transfer to the intensive care unit, mean systemic arterial pressures were found to be low, with transient reduction to as low as 40 mmHg when the patient coughed or spoke. Transthoracic echocardiography (Table 2) showed a dilated right ventricle with globally reduced systolic function. Right ventricular systolic pressures were shown to be 170 mmHg. Milrinone and epinephrine were started for hemodynamic support.

Due to progressive symptomatic and hemodynamic deterioration, he was transferred to the Cardiovascular Intensive Care Unit under the cardiac surgery service at another hospital to facilitate Novalung insertion via central cannulation. The goal was to decompress the right ventricle in order to stabilize the patient's hemodynamics, as well as bridge to diagnosis and possibly lung transplant assessment.

In the operating room, the patient arrested when anesthetic induction was attempted. Thus, he was bridged to cardiopulmonary bypass via cardiac massage. Venous and arterial cannulation of the Novalung were, respectively, achieved via the pulmonary artery and left atrium through Sondergaard's groove, with simultaneous surgical lung biopsy. Novalung was started on 2 L sweep gas at 2 L flow. After sternal closure, the patient became increasingly hypotensive with transesophageal echocardiography showing right ventricular dysfunction. Thus, the patient was reopened and cardiopulmonary bypass was reinitiated. With extensive vasoplegia and increased demand for vasoconstrictors, cardiopulmonary bypass was converted to venoarterial ECMO to maintain mechanical support. At the end of the procedure, the sternum was left open with slight retraction provided by a converted 20 mL syringe, and the skin was closed.

**Figure 2:**
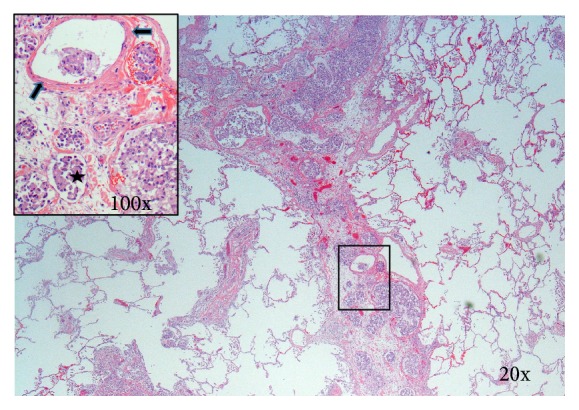
Section of postmortem lung tissue demonstrating thickened edematous interlobular septum with numerous dilated lymphatic channels filled with malignant glandular cells (starred in inset). Inset also shows tumor cells in venous channels with thicker walls (arrows). Hematoxylin and eosin.

**Figure 3:**
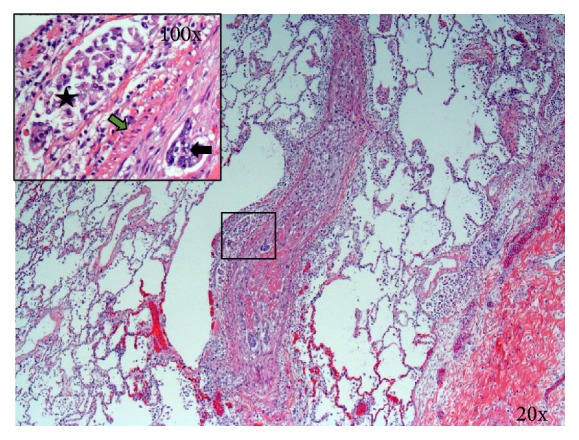
Section of lung showing diffusely thrombosed artery with recanalization and small focus of intra-arterial malignant cells (arrow). Inset also shows adjacent lymphatic channel with malignant cells (star). Green arrow points to the muscular wall of the artery. Hematoxylin and eosin.

**Figure 4:**
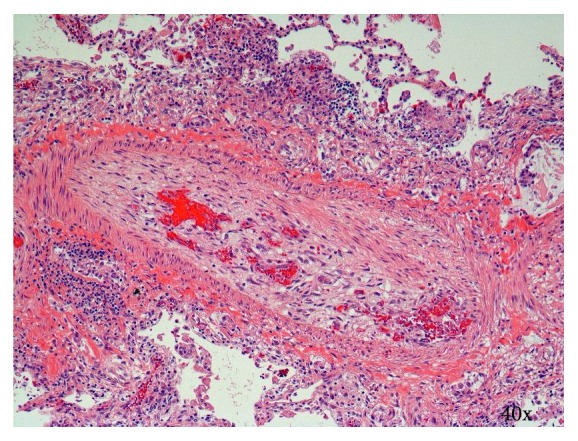
Section showing thrombosed medium-sized pulmonary artery with recanalization. Various stages of thrombosis were observed in many arteries throughout all lobes. Hematoxylin and eosin.

Postoperatively, the patient was maintained on VA-ECMO and Novalung for 110 h (Table 2). Flows were maintained at 2.5–3 L/min and 2.5–3.5 L/min for VA-ECMO and Novalung, respectively. On postoperative day 2, the surgical lung biopsy demonstrated signet-ring cell pulmonary tumor embolism and lymphangitic carcinomatosis. Figures 2 and 3 depict histological sections demonstrating lymphangitic carcinomatosis of the lung and tumor thrombus in both arterial and venous pulmonary vasculatures.  Figure 4 demonstrates a thrombosed pulmonary artery, explaining the subacute pulmonary hypertension. Given the extent of metastatic infiltration and the need for mechanical support, palliative chemotherapy was contraindicated. After discussion with the family, all modes of support were withdrawn in appropriate order and the patient passed away.

Autopsy results supported the diagnosis of lymphangitic carcinomatosis with signet-ring cell morphology. Vascular features suggested grades 3 and 4 pulmonary hypertension and multiple tumor thrombi. Right ventricle of the heart was moderately dilated. Examination of the stomach revealed a nonperforated 3.3 × 2 cm ulcer with involvement of poorly differentiated signet-ring cell adenocarcinoma. All sampled perigastric, peripancreatic, mesenteric, and omental nodules were positive for metastatic adenocarcinoma.

## 3. Discussion

Extracorporeal membrane oxygenation was first reported as a long-term bridge to recovery in 1972 [1], to transplant in 1991 [2], and to left ventricular assist devices in 1999 [10]. The use of centrally cannulated Novalung pECLA was first reported in pulmonary arterial hypertension and bridge to transplant in 2009 [11]. These modalities of ECLS are indicated in respiratory or right ventricular failure refractory to medical management, both in potentially reversible conditions and in irreversible conditions in transplant candidates [12].

Signet-ring cell gastric carcinoma is a rare tumor that is increasing in incidence in an era of risk modification for other forms of gastric adenocarcinoma. In advanced stages, this tumor is highly infiltrative and is less chemosensitive compared to other gastric adenocarcinomas. The rarity of the tumor and the nonspecific presentation of pulmonary tumor embolism were both factors in the difficulty of clinical diagnosis [13].

In this case, the rapid progression of disease and the etiology and reversibility of the patient's respiratory failure were uncertain. The acuity of respiratory and hemodynamic failure prevented our team from employing crucial diagnostic investigations, including ventilation-perfusion scans and CT angiography, both previously shown to aid in the diagnosis of PTE [14–16]. Most importantly, a tissue diagnosis from a surgical lung biopsy was not feasible due to the deteriorating clinical state. ECLS bridging facilitated these investigations, allowing for informed decision-making by both the clinical team and the patients family.

## 4. Conclusion

Our experience adds to the growing body of knowledge regarding the use of ECLS modalities including VA-ECMO and centrally cannulated Novalung pECLA as a bridge to diagnosis in unstable patients [1, 17, 18]. This approach facilitates good decision-making regarding withdrawal of support, bridge to recovery, or bridge to transplantation. We advocate this approach in diagnostically uncertain cases in unstable patients who are otherwise good ECLS and transplant candidates.

## Figures and Tables

**Table 1:** Patient characteristics.

Parameter	Characteristic
Age (y)	38
Gender	Male
Weight (kg)	86.9
Height (m)	1.8
BMI (kg/m^2^)	26.8
Presentation	Respiratory failure
Procedure	Central cannulation ECLS
	Open lung biopsy

**Table 2:** Surgical data.

Parameter	Value
*Preoperative echocardiography*	
Right ventricular dimensions (mm)	
Annulus	52
Mid-cavity	60
Longitudinal	75
Free wall thickness	8
Tricuspid annular plane systolic excursion (mm)	<10 mm
Right ventricular systolic pressure (mmHg)	170
Mean pulmonary artery pressure (mmHg)	100

*Perioperative*	
Total cardiopulmonary bypass time (min)	164

*Postoperative*	
Total ECLS time	110
VA-ECMO flow (L/min)	2.5–3
Novalung flow (L/min)	2.5–3.5
Final diagnosis	Pulmonary tumor embolism
	Signet-ring cell morphology
Status	Deceased
